# The Rational Treatment of Running Ears

**Published:** 1905-10-21

**Authors:** F. Faulder White


*To the Editor of* The Hospital.

### THE RATIONAL TREATMENT OF RUNNING EARS.

*To the Editor of* The Hospital.

Dear Sir,—I am obliged for the notice of my little book in last week's issue of The Hospital. May I say a few words in reference to my views of the radical operation? I have always recognised that in certain cases a radical operation may give the best chance, but I dissent from much of the teaching of the present day, which, making light of the special dangers to life, features, and hearing that a radical operation involves, advises its performance in many conditions which I have found will yield to measures decidedly less dangerous. Disinfection of the ear can almost always be accomplished by intra-meatal treatment unless the bone be deeply implicated. Otectomy I have found after considerable experience to be free from danger to life, features, and hearing. I am, Sir, yours truly,

F. Faulder White.

*** We quite agree that the radical mastoid operation is sometimes performed where milder measures would suffice. But we think that the same criticism applies also to intra-meatal operative procedures.—Ed. The Hospital.

